# Ferroptosis is involved in deoxynivalenol-induced intestinal damage in pigs

**DOI:** 10.1186/s40104-023-00841-4

**Published:** 2023-03-16

**Authors:** Meng Liu, Lei Zhang, Yixin Mo, Jiahuan Li, Jiacheng Yang, Juan Wang, Niel Alexander Karrow, Hao Wu, Lvhui Sun

**Affiliations:** 1 State Key Laboratory of Agricultural Microbiology, Hubei Hongshan Laboratory, Frontiers Science Center for Animal Breeding and Sustainable Production, College of Animal Sciences and Technology, Huazhong Agricultural University, Wuhan, Hubei 430070 China; 2 Newhope Liuhe Co. Ltd., Beijing, 100102 China; 3 Department of Animal Biosciences, University of Guelph, Guelph, ON N1G2W1 Canada

**Keywords:** Deoxynivalenol, Ferroptosis, Intestine, Piglets, Toxicity

## Abstract

**Background:**

Deoxynivalenol (DON) is a widespread issue for feed and food safety, leading to animal and human health risks. The objective of this study was to determine whether ferroptosis is involved in DON-induced intestinal injury in piglets. Three groups of 21-day-old male weanling piglets (*n* = 7/group) were fed a control diet, or diet adding 1.0 or 3.0 mg DON/kg. At week 4, serum and small intestines were collected to assay for biochemistry, histology, redox status and ferroptosis-related genes expression. In addition, the involvement of ferroptosis and the role of *FTL* gene in DON-induced cell death were further verified in the IPEC-J2 cells.

**Results:**

Compared to the control, dietary supplementation of DON at 1.0 and 3.0 mg/kg induced different degrees of damage in the duodenum, jejunum and ileum, and increased (*P* < 0.05) serum lipopolysaccharide concentration by 46.2%–51.4%. Dietary DON supplementation at 1.0 and (or) 3.0 mg/kg increased (*P* < 0.05) concentrations of malondialdehyde (17.4%–86.5%) and protein carbonyl by 33.1%–92.3% in the duodenum, jejunum and ileum. In addition, dietary supplemented with DON upregulated (*P* < 0.05) ferroptotic gene (*DMT1*) and anti-ferroptotic genes (*FTL* and *FTH1*), while downregulated (*P* < 0.05) anti-ferroptotic genes (*FPN*, *FSP1* and *CISD1*) in the duodenum of the porcine. Furthermore, the in vitro study has demonstrated that deferiprone, a potent ferroptotic inhibitor, mitigated (*P* < 0.05) DON-induced cytotoxicity in porcine small intestinal IPEC-J2 cells. Additionally, deferiprone prevented or alleviated (*P* < 0.05) the dysregulation of ferroptosis-related genes (*ACSL4* and *FTL*) by DON in IPEC-J2 cells. Moreover, specific siRNA knockdown *FTL* gene expression compromised the DON-induced cell death in IPEC-J2 cells.

**Conclusions:**

In conclusion, this study revealed that ferroptosis is involved in DON-induced intestinal damage in porcine, and sheds a new light on the toxicity of DON to piglets.

**Supplementary Information:**

The online version contains supplementary material available at 10.1186/s40104-023-00841-4.

## Introduction

Deoxynivalenol (DON), is a type B trichothecene largely generated by *Fusarium graminearum* and *F. culmorum*. DON is one of the most widespread mycotoxins contaminates in cereal, including wheat, barley, oats, millet and corn and their by-products [1–3]. Climate change and associated global warming is increasing crop susceptibility to fungal infection, which is further resulting in increased DON contamination of cereals [3]. It was reported that consumption of food and feed contaminated by DON leads to food refusal, emesis, diarrhea and impaired intestinal and immune function in humans and farm animals [4–6]. It also could be residual in the animal food as the secondary environmental pollution to endanger human health [6, 7]. Therefore, in view of the harmful effects of DON, its toxic mechanisms to animals have drawn great attention during the past decades [8].

DON is quickly and efficiently absorbed in the upper part of the small intestine, which is the primary target organ damaged by DON [9]. It is well documented that the intestinal toxicity of DON is associated with impairment of the intestinal structure, epithelial barrier, intestinal mucosal immunity and gut microbiota homeostasis [2, 10]. These DON-induced intestinal damages have been attributed primary to DON’s ability to binding to the eukaryotic 60S ribosomal subunit, blocking peptidyl transferase, and inhibiting translation, but concurrently activating mitogen-activated protein kinase via the “ribotoxic stress response” and inducing inflammation and apoptosis [11]. Also, generation of DON-induced reactive oxygen species (ROS) can induce oxidative stress and apoptosis, which has been recognized as another outcome of DON toxicity [10, 12].

Unlike apoptosis, ferroptosis is a newly discovered form of iron-dependent and ROS-reliant cell death with characteristics of lipid peroxide accumulation, and cytological change, including reduced or vanished mitochondrial cristae, and ruptured and condensed mitochondrial membrane [13]. However, whether ferroptosis is involved in DON-induced intestinal damage remains unclear. Among animal species, pigs are highly susceptible to DON [14]. Thus, in this study, pigs were selected to determine whether or not DON-induced intestinal injury is associated with the regulation of ferroptosis signaling.

## Materials and methods

### Piglets, treatments, and sample collection

The animal protocol for this study was approved by the Institutional Animal Care and Use Committee of Huazhong Agricultural University, China. In total, 21 castrated male crossbred [(Duroc × Landrace) × Large White] weanling piglets (aged 3 weeks) were randomly allocated to 3 groups; each group was assigned to 7 pens of 1 piglet/pen. Piglets were allowed free access to water and a corn-soybean based diet (Control; Additional file 1: Table S1**)** formulated to meet the NRC nutritional requirements (NRC, 2012) [15], or the control diet spiked with 1.0 mg/kg DON or 3.0 mg/kg DON. Based on the Chinese hygiene standard of 1.0 mg/kg for DON in pig compound feed and since it has been reported that diet contaminated with 2.89 mg/kg DON decreased the performance and caused intestinal damage in piglets, we set the above doses of DON [16]. The DON was produced by the *Fusarium graminearum* strain W3008 and mixed into the pig feed as in our previous study [16]. The concentrations of DON, aflatoxin B_1_ and zearalenone in the feed were measured by specific assay kits (COKAS4000W, COKAQ8000 and COKAS5000W) from the Romer Labs, Singapore. The experiment lasted 28 d. At the end of the experiment, all pigs were humanely euthanized by intravenous injection of sodium pentobarbital (40 mg/kg BW) to harvest blood and intestine for either serology analysis or histology examination. Five-cm of mid-duodenum, mid-jejunum and mid-ileum were cut and washed with ice-cold saline, then divided into aliquots and collected in Eppendorf tubes that were snap-frozen in liquid nitrogen and stored at −80 °C until use.

### Histology, serum biochemistry and redox status analysis

The duodenum, jejunum, ileum tissues were microscopically examined after fixing in 10% neutral-buffered formalin and processing for paraffin embedding, sectioning at 5 μm, and then staining with hematoxylin and eosin [17]. The concentrations of lipopolysaccharide (LPS), malondialdehyde (MDA), protein carbonyl (PC) and reduced glutathione (GSH) and activity of diamine oxidase (DAO), superoxide dismutase (SOD), total antioxidant capacity (T-AOC) were measured by a colorimetric method with the use of specific assay kits (H255, A003-1-2, A087-1-2, A006-1-1, A088-1-1, A001-1-2 and A015-1-2) from the Nanjing Jiancheng Bioengineering Institute of China. Protein concentration was measured by the bicinchoninic acid assay (Beyotime Institute of Biotechnology, Jiangsu, China).

### Porcine IPEC-J2 cell culture and viability assay

The porcine IPEC-J2 cell line was cultured following the method as previously described [16]. Briefly, the cells were grown in DMEM supplemented with 10% fetal bovine serum (FBS), 100 IU penicillin and 100 mg/mL streptomycin at 37 °C in a humidified 5% CO_2_ atmosphere, and medium was changed at 24 h intervals. For DON exposure, IPEC-J2 cells were seeded in a 96-well plate (1 × 10^4^ cells/well) and incubated for 24 h, then, treated with DON (0–1000 μg/L; Additional file 2: Fig. S1A), or deferiprone (DFP, 10-80 μmol/L; Additional file 2: Fig. S1B) for 24 h to determine the 30% inhibitory concentration (IC_30_) of DON or the safety dose of DFP by measuring cells viability with the CCK-8 kit following the manufacturer’s instruction (Beyotime biotechnology, China). The determined IC_30_ of DON and safety dose of DFP were 500 μg/L and 20 μmol/L, respectively, which were used for the further analysis. 1) To verify whether ferroptosis involved in DON-induced cell death in IPEC-J2 cells, the cells were treated with normal cell cultural medium (control), control added with 500 μg/L DON (DON), or control added with 500 μg/L DON plus 20 μmol/L DFP (DON+DFP) for 24 h. Then, calcein acetoxymethyl ester (Calcein AM, Abcam, ab141420) were used for assaying the cells viability. The fluorescence intensity was observed and analyzed using fluorescence microscope and software Image J. 2) To verify whether ferritin light chain (*FTL*) involved in DON-mediated ferroptosis in IPEC-J2 cells, the cells were treated with negative control siRNA (NC), negative control siRNA plus 500 μg/L DON (NC+DON), 50 nmol/L *FTL* siRNA (siRNA), or 50 nmol/L *FTL* siRNA plus 500 μg/L DON (siRNA+DON). Briefly, the siRNA (Additional file 3: Table S2) was transfected to the cells with 0.2% Lipofectamin 2000 reagent (Invitrogen, Shanghai, China) treatment for 48 h incubation, then, 500 μg/L DON was added for 24 h treatment. Then, the cells viability was measured by CCK-8 kit as described above. The cells were also collected and stored at −80 °C until other assays.

### Real-time q-PCR and western blot analyses

Real-time q-PCR analysis was conducted as previously described [5]. Briefly, total mRNA was extracted from the jejunum with Trizol (Invitrogen) following the instructions of the manufacturer. Primers for ferroptosis-related genes and the house keeping gene β-actin were designed using Primer Express 3.0 (Applied Biosystems) and are presented in Additional file 4: Table S3. The 2^-ddCt^ method was used for the quantification of target genes, and the relative abundance of target genes was normalized to β-actin. Western blot analyses of the jejunum samples were performed as previously described [18], and the primary antibody used for each gene is presented in Additional file 5: Table S4. The concentration of protein was detected by the bicinchoninic acid assay (Beyotime Institute of Biotechnology, Jiangsu, China).

### Statistical analysis

Statistical analysis was performed with the SPSS (version 13, Chicago, IL, USA). Data were analyzed by a one-way ANOVA with a significance level of *P* < 0.05, and the Tukey-Kramer method was used for multiple mean comparisons. Data are presented as means ± SD.

## Results

### Intestinal histology, serum biochemistry, and redox status

**Fig. 1:**
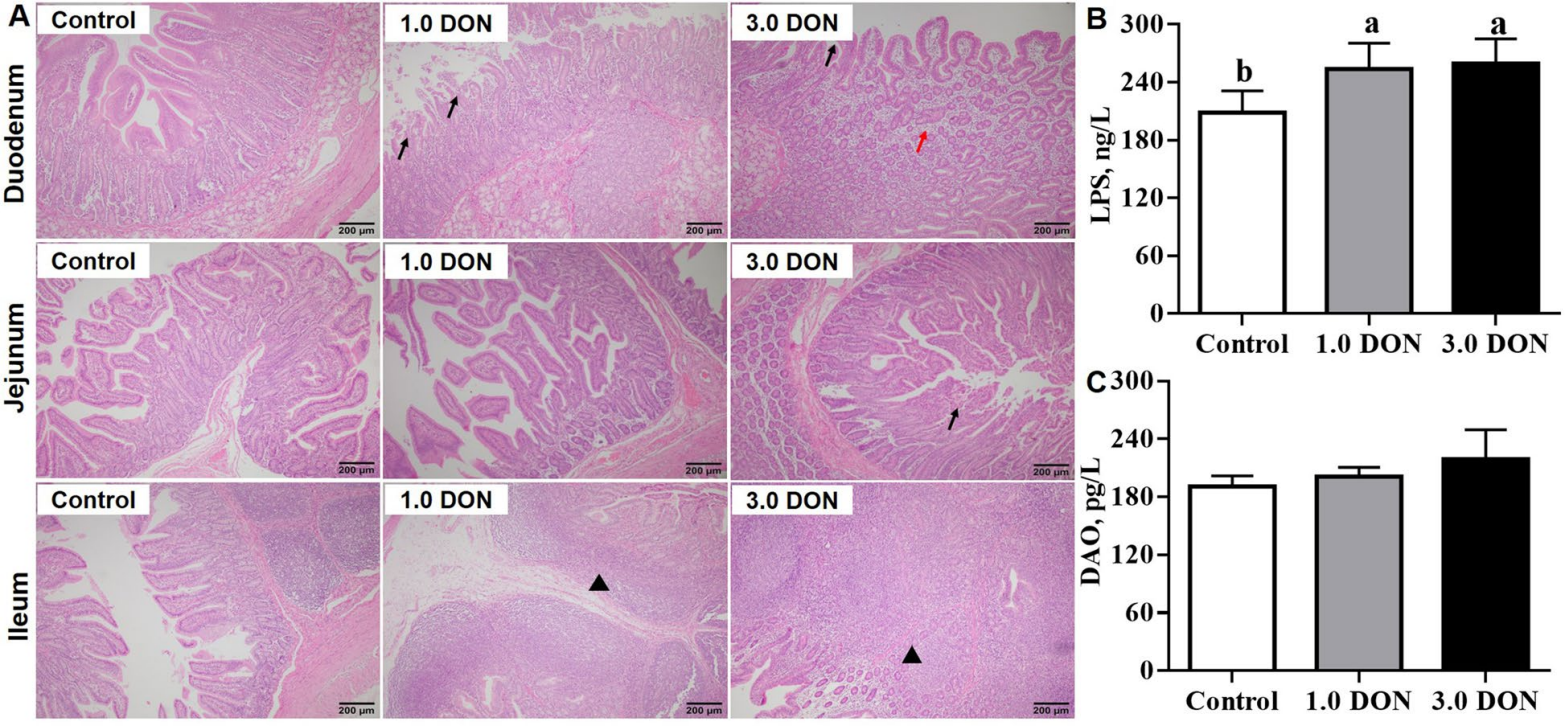
Effects of DON on histology of intestine and serum biochemistry in weaned piglets. Histological sections of duodenum, jejunum and ileum (**A**). The content of LPS (**B**) and DAO (**C**) in serum. Values are expressed as means ± SD, *n* = 7. The sections were stained with hematoxylin and eosin; photomicrographs are shown at 200× magnification. Black arrow indicates degeneration, necrosis and desquamation of villous epithelial cells; Black arrowhead indicates lymphocyte hyperplasia; Red arrow indicates lamina propria edema. Labeled means without a common letter differ, *P <* 0.05. LPS, lipopolysaccharid, DAO, diamine oxidase; Control, base diet; 1.0 DON, basal diet supplemented with 1.0 mg/kg DON; 3.0 DON, basal diet supplemented with 3.0 mg/kg DON

**Fig. 2:**
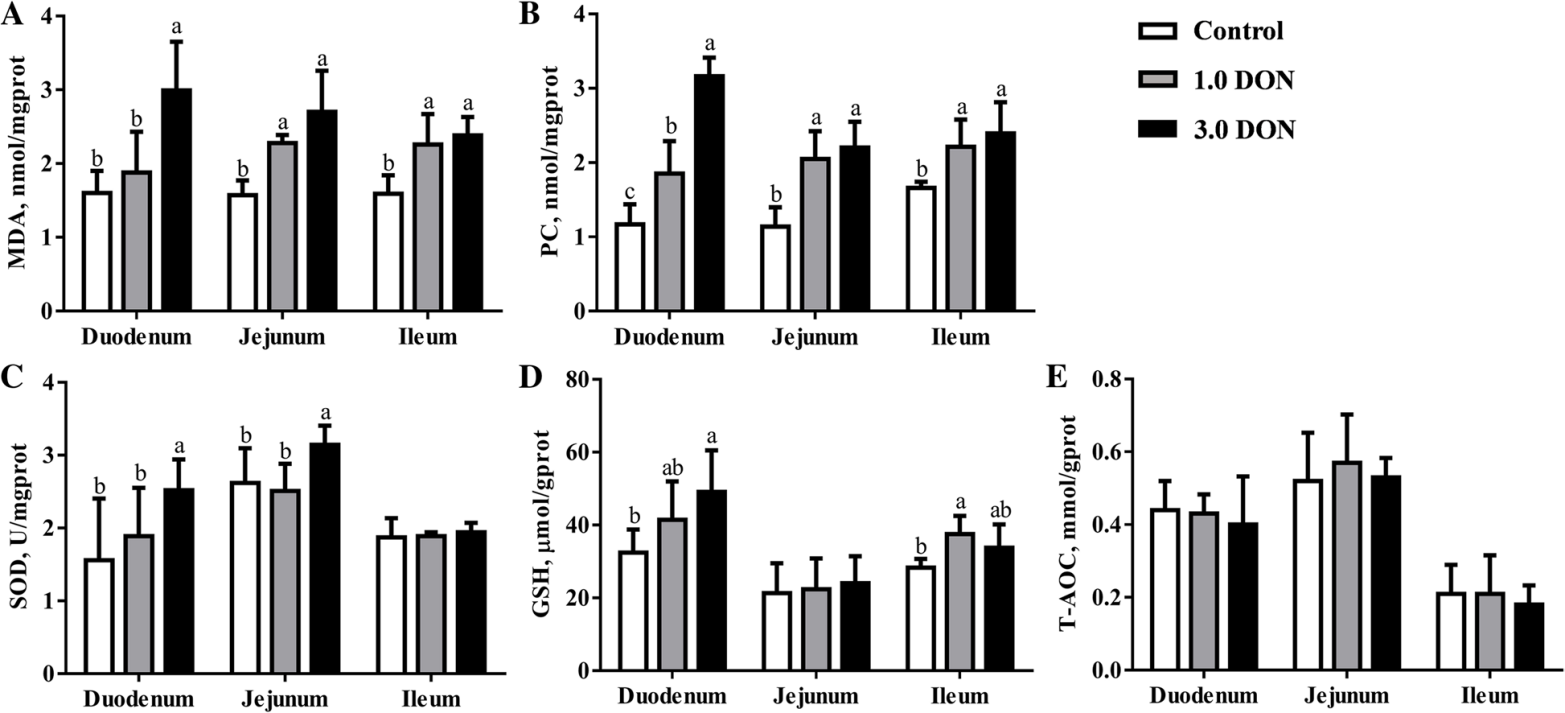
Effects of DON on redox status of intestine in weaned piglets. Values are expressed as means ± SD, *n* = 7. Labeled means without a common letter differ, *P <* 0.05. MDA, malondialdehyde; PC, protein carbonyl, SOD, superoxide dismutase; GSH, reduced glutathione; T-AOC, total antioxidant capacity; Control, base diet; 1.0 DON, basal diet supplemented with 1.0 mg/kg DON; 3.0 DON, basal diet supplemented with 3.0 mg/kg DON

As shown in Fig. 1A, compared with the control, dietary supplementation of DON at 1.0 and (or) 3.0 mg/kg induced degeneration and necrosis of villous epithelium cell, and lamina propria edema in duodenum, degeneration and necrosis of villous epithelium cell in the jejunum, lymphocyte hyperplasia in ileum. Meanwhile, dietary supplementation of DON at 1.0 and 3.0 mg/kg increased (*P* < 0.05) the LPS concentration by 46.2% and 51.4% in the serum of piglets (Fig. 1B), while DON did not affect (*P* ≥ 0.05) the DAO activity in the serum (Fig. 1C). Compared with the control, dietary supplementation of DON at 1.0 mg/kg increased (*P* < 0.05) concentrations of MDA by 41.9% and 45.5% in the jejunum and ileum, while 3.0 mg/kg DON increased (*P* < 0.05) concentrations of MDA by 49.1%–86.5% in the duodenum, jejunum and ileum (Fig. 2A). In addition, dietary supplementation of DON at 1.0 increased (*P* < 0.05) concentrations of PC by 33.1%–79.8% and 3.0 mg/kg DON increased (*P* < 0.05) concentrations of PC by 43.9%–170.0% (Fig. 2B) in the duodenum, jejunum and ileum. Meanwhile, dietary supplementation of DON at 3.0 mg/kg increased (*P* < 0.05) SOD activity in the duodenum and jejunum by 19.8%–61.5% and dietary supplementation of DON at 1.0 mg/kg increased (*P* < 0.05) GSH concentration by 32.5% in the ileum (Fig. 2C and D). However, dietary supplementation of DON did not affect (*P* ≥ 0.05) the T-AOC in the intestinal samples (Fig. 2E).

### Expression of ferroptosis-related genes in duodenum

**Fig. 3:**
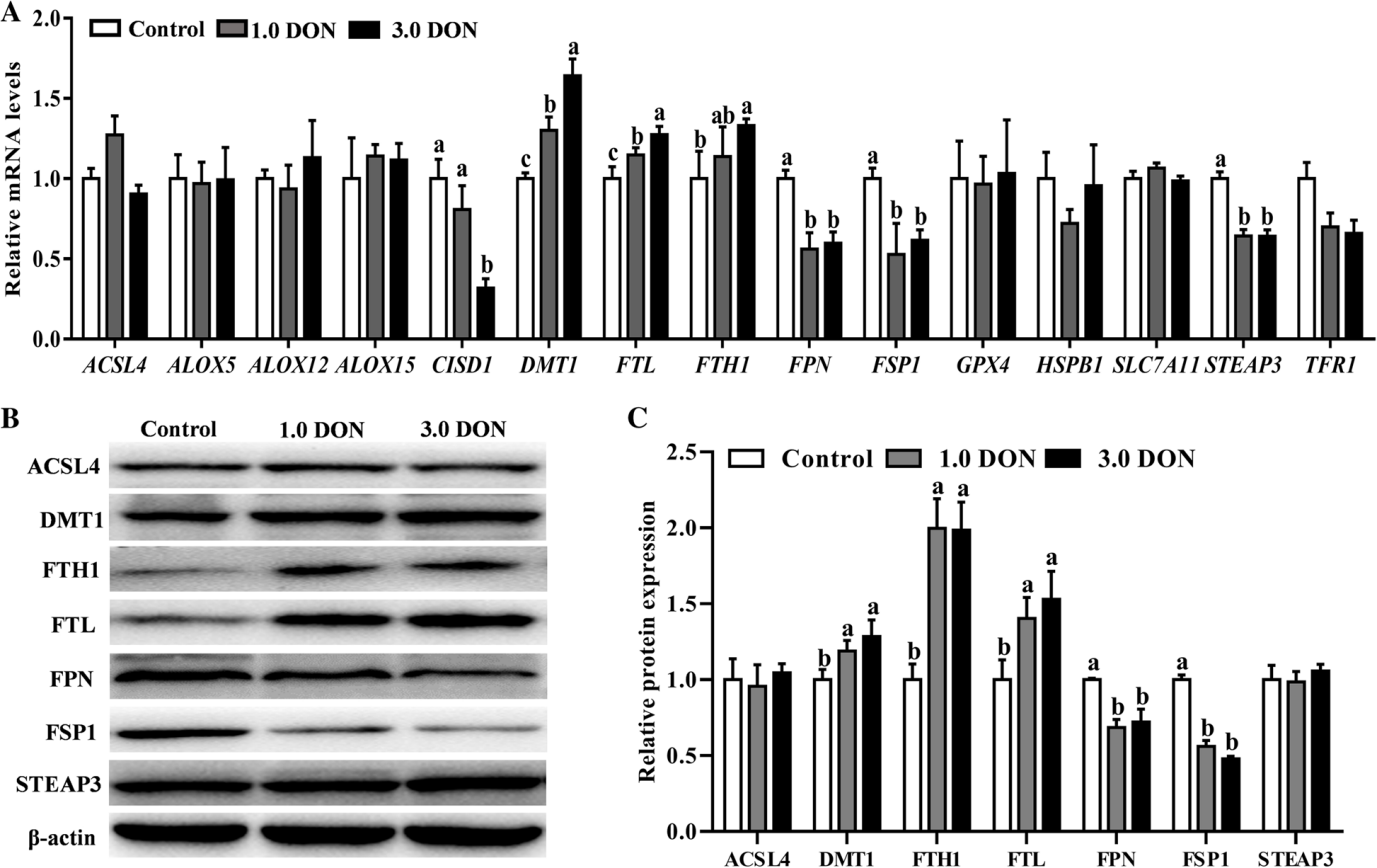
Effects of DON on the expression of ferroptosis-related genes in duodenum. The relative mRNA abundance of ferroptosis-related genes in duodenum (**A**). Values are expressed as means ± SD, *n* = 7. A representative image (**B**) and the relative density (**C**) of protein bands of ferroptosis-related proteins in duodenum. Values are expressed as means ± SD, *n* = 3. Labeled means without a common letter differ, *P <* 0.05. Control, base diet; 1.0 DON, basal diet supplemented with 1.0 mg/kg DON; 3.0 DON, basal diet supplemented with 3.0 mg/kg DON. *ACSL4*, acyl-CoA synthetase long chain family member 4; *ALOX5*, arachidonate 5-lipoxygenase; *ALOX12*, arachidonate 12-lipoxygenase, 12S type; *ALOX15*, arachidonate 15-lipoxygenase; *CISD1*, CDGSH iron sulfur domain 1; *DMT1*, divalent metal transporter 1; *FTL*, ferritin light chain; *FPN*, ferroportin; *FTH1*, ferritin heavy chain 1; *FSP1*, ferroptosis suppressor protein 1; *GPX4*, glutathione peroxidase 4; *HSPB1*, heat shock protein family B (small) member 1; *SLC7A11*, solute carrier family 7 member 11; *STEAP3*, six-transmembrane epithelial antigen of prostate 3; *TFR1*, transferrin receptor

The expressions of 15 ferroptosis-related genes at mRNA and (or) protein levels in the duodenum are presented in Fig. 3. Specifically, both dietary supplementation of DON at 1.0 mg/kg and 3.0 mg/kg increased (*P* < 0.05) the mRNA levels of divalent metal transporter 1 (*DMT1*) and *FTL* and decreased (*P* < 0.05) ferroportin (*FPN*), ferroptosis suppressor protein 1 (*FSP1*) and six-transmembrane epithelial antigen of prostate 3 (*STEAP3*). Notably, dietary supplementation of DON at 3.0 mg/kg also increased (*P* < 0.05) the mRNA levels of ferritin heavy chain 1 (*FTH1*) and decreased (*P* < 0.05) CDGSH iron sulfur domain 1 (*CISD1*) in the duodenum (Fig. 3A). Furthermore, dietary supplementation of DON at 1.0 and 3.0 mg/kg also increased (*P* < 0.05) DMT1, FTH1 and FTL and decreased (*P* < 0.05) FPN and FSP1 at protein levels in the duodenum (Fig. 3B and C). However, the expression of the rest of 8 genes was not significantly affected (*P* ≥ 0.05) by the DON supplementation in the duodenum of porcine (Fig. 3A–C).

### Verification of ferroptosis involvement in DON-induced cell death in IPEC-J2 cells

**Fig. 4:**
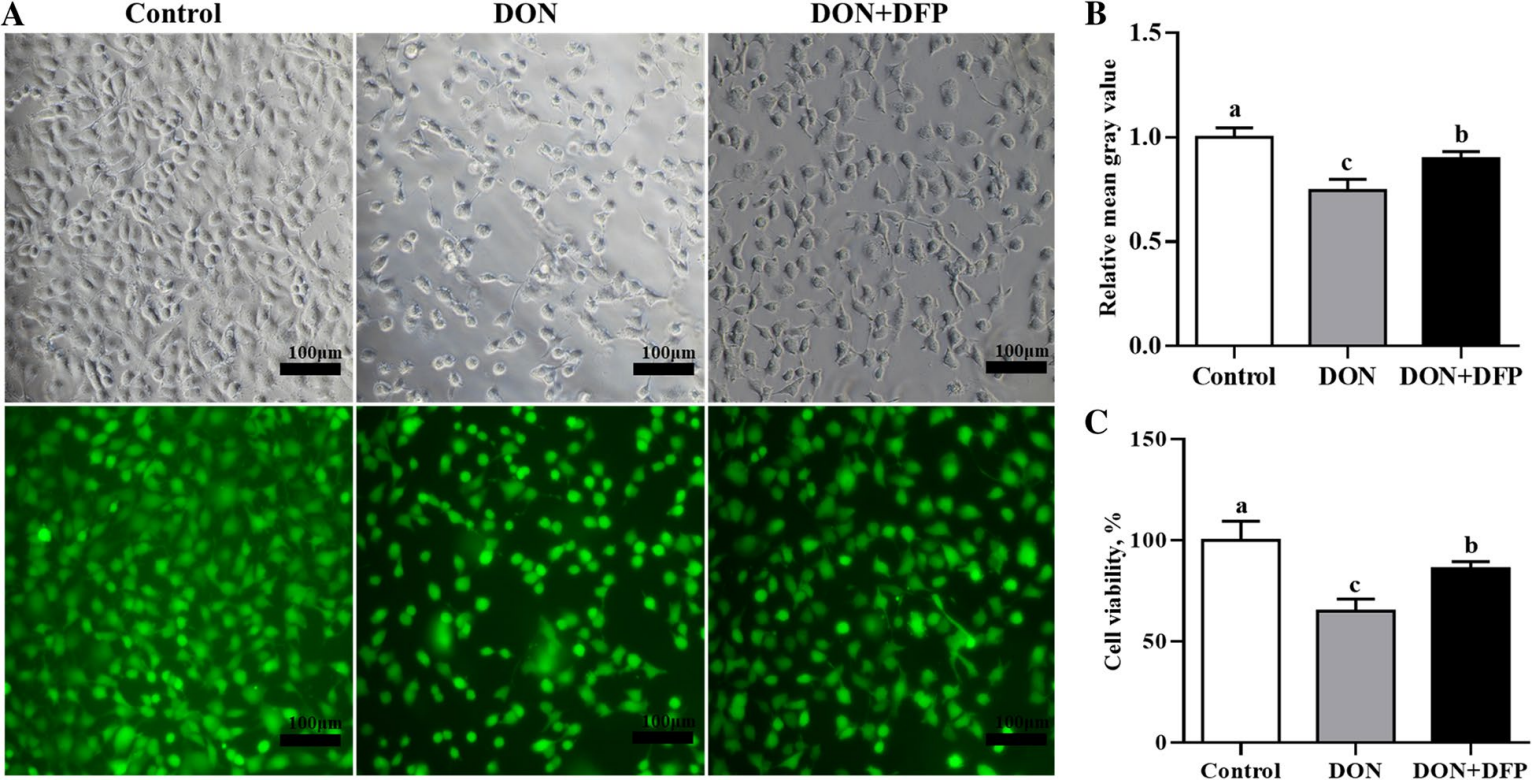
Effects of DON and DFP on IPEC-J2 cell viability. The cells viability was assayed by calcein acetoxymethyl ester (Calcein AM; **A**) and the values shows the fluorescence intensity was analyzed by Image J (**B**). Cell viability was analyzed by CCK-8 (**C**). Values are expressed as means ± SD, *n* =6. Labeled means without a common letter differ, *P <* 0.05. Control, cell culture medium; DON, cell culture medium+500 μg/L DON; DON+DFP, cell culture medium+500 μg/L DON+20 μmol/L DFP

**Fig. 5:**
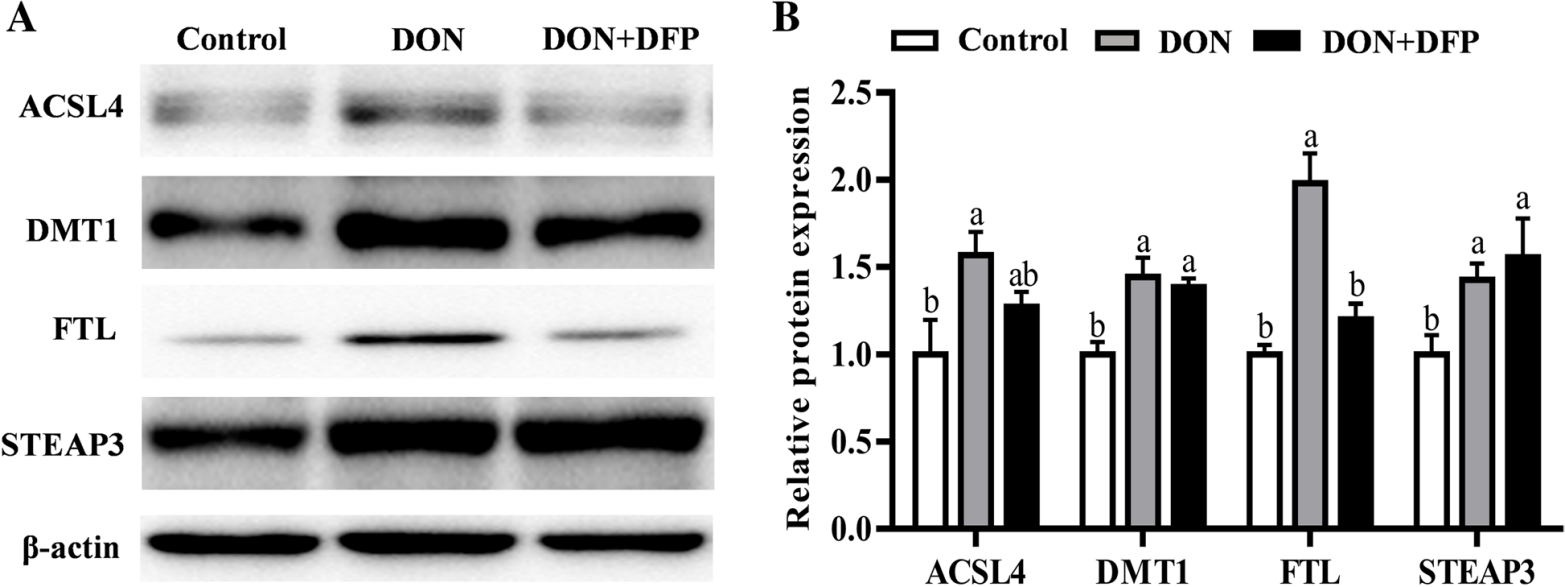
Effects of DON and DFP on the expression of ferroptosis-related proteins in IPEC-J2. A representative image (**A**) and the relative density (**B**) of protein bands of ferroptosis-related proteins in IPEC-J2 cells. Values are expressed as means ± SD, *n* = 3. Labeled means without a common letter differ, *P <* 0.05. ACSL4, acyl-coenzyme A synthetase long-chain family member 4; DMT1, metal transporter 1; FTL, ferritin light chain; STEAP3, six-transmembrane epithelial antigen of prostate 3. Control, cell culture medium; DON, cell culture medium+DON; DON+DFP, cell culture medium+ DON+DFP

Compared with the control, DON supplementation reduced (*P* < 0.05) the IPEC-J2 cell viability (Fig. 4A–C) by 25.3% and 34.5%, as evidenced by the Calcein AM staining and CCK-8 analysis. Notably, DON-induced changes were alleviated (*P* < 0.05) by 15.1% and 20.5% in the IPEC-J2 cells by the supplementation with DFP (Fig. 4A–C). Furthermore, DON supplementation upregulated (*P* < 0.05) acyl-coenzyme A synthetase long-chain family member 4 (ACSL4), DMT1, FTL and STEAP3 protein productions compared with the control (Fig. 5A and B). Notably, changes of the ACSL4 and FTL protein productions observed in the DON group were attenuated (*P* < 0.05) in the DON+DFP group (Fig. 5A and B**)**.

### Verification of the role of FTL involvement in DON-mediated ferroptosis in IPEC-J2 cells

**Fig. 6:**
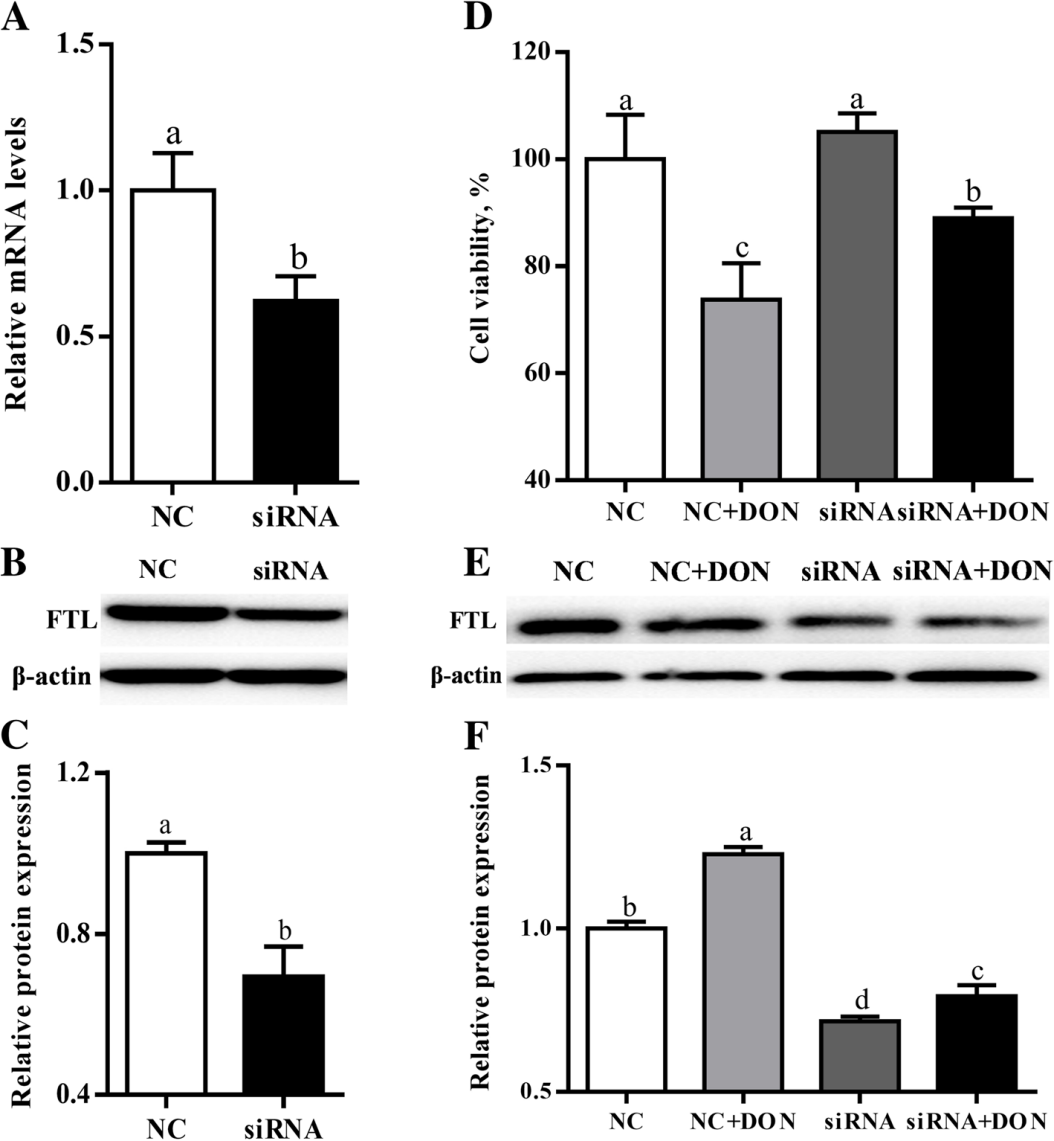
Verification of the role of FTL in involvement in DON-mediated ferroptosis in IPEC-J2 cells. Relative mRNA (**A**), a representative image (**B**) and the relative density (**C**) of protein bands of FTL in IPEC-J2 cell after *FTL* siRNA transfection. IPEC-J2 cell viability was analyzed by CCK-8 after treated by *FTL* siRNA transfection and DON treatment (**D**). A representative image (**E**) and the relative density (**F**) of protein bands of FTL in IPEC-J2 cell after *FTL* siRNA transfection and DON treatment. Values are expressed as means ± SD, *n* = 3–6. Labeled means without a common letter differ, *P <* 0.05. NC, cells treated with negative control siRNA; siRNA, cells treated with *FTL* siRNA; NC+DON, cell treated with negative control siRNA plus DON; siRNA+DON, cell treated with *FTL* siRNA plus DON

Compared with the control, the IPEC-J2 cells treated with *FTL*-specific siRNA had downregulated (*P* < 0.05) *FTL* expression at mRNA level (Fig. 6A). Furthermore, the *FTL*-specific siRNA treatment also downregulated (*P* < 0.05) FTL protein production by IPEC-J2 cells (Fig. 6B and C). Compared with the control, DON supplementation reduced (*P* < 0.05) the viability of IPEC-J2 cells by 26.2%, while knockdown of *FTL* mitigated (*P* < 0.05) these changes induced by DON (Fig. 6D). Additionally, DON supplementation upregulated (*P* < 0.05) FTL protein production by IPEC-J2 cells compared with the control (Fig. 6E and F). Notably, upregulation of the FTL protein production observed in the DON group was prevented (*P* < 0.05) in the DON+siRNA group (Fig. 6E and F**)**.

## Discussion

Dietary supplementation of DON at 1.0 and 3.0 mg/kg was shown to induce intestinal damage. Piglets that consumed DON manifested pathological signs of intestinal injury, including degeneration, necrosis and lymphocyte hyperplasia, and (or) lamina propria edema in duodenum, jejunum and ileum. These outcomes were in agreement with previous studies, which have reported that pigs fed diets contaminated with 2.89 and 4.0 mg/kg DON exhibited gastrointestinal damage [16, 19, 20]. In addition, LPS is a pivotal virulence factor and present in the outer membrane of Gram-negative bacteria [21]; increased gut permeability or damage can be recognized by the leakage of LPS into the blood [22, 23]. Our results indicate a higher serum LPS concentration for DON supplementation than for the control, which would seem to confirm an impairment of the intestinal integrity and injury. Notably, although the Chinese safety standard sets 1.0 mg/kg for DON in pig compound feed [3], the current study showed that 1.0 mg/kg of DON had caused significant damage to the gastrointestinal tract of piglets. These findings warn us that suitable remediation strategy for DON need to be applied in the feed industry.

Impairment of redox balance is well-documented as one of the common mechanisms for DON-triggered cell deaths in different organs of animals [24–26]. Indeed, the piglets exposed to 1.0 and 3.0 mg/kg DON suffered from intestinal oxidative stress, as indicated by increase of the biomarkers of lipid oxidation (MDA) and protein oxidation (PC) to varying degrees in duodenum, jejunum and ileum. Intriguingly, SOD and GSH, which play pivotal roles in the antioxidant defense, were partly increased by DON in duodenum, jejunum and (or) ileum in the current study. This might be explained as a compensatory mechanism that piglets activated the antioxidant system as an adaptation to the DON-induced oxidative damage in intestine [27]. This helps piglets to maintain the redox homeostasis under DON-induced damage in intestine [27]. These outcomes are in agreement with previous reports, which reported that 3.0 mg/kg and 10 μmol/L DON impaired the redox homeostasis in mice and human intestinal cell line Caco-2 [28–30]. Taken together, these results implicate that DON-induced oxidative stress as the cause of cell death could be one of the major reasons for the intestinal damage [21–33].

An interesting finding from the present study is that dysregulation of ferroptosis signaling expression appears to be a novel mechanism for the DON-induced intestinal injury damage in piglets. Specifically, dietary DON supplementation upregulated of DMT1, FTL and FTH1, and downregulated FPN, FSP1 and *CISD1* at mRNA and (or) protein levels in the duodenum. Because DMT1 is responsible for Fe^2+^ import, which would result in lipid peroxidation and ferroptosis, and FPN is responsible for the Fe^2+^ export, which plays roles in inhibiting ferroptosis [34], both FSP1 and CISD1 can protect against mitochondrial lipid peroxidation, and thus inhibit ferroptosis [35, 36]. The upregulation of DMT1 and downregulation of FPN, FSP1 and *CISD1* by DON may induce ferroptosis in this study. Ferritin, a protein complex represented by FTL and FTH1, plays roles in cytoplasmic iron storage and contributes to inhibiting ferroptosis [37]. Strikingly, these two proteins were upregulated by DON in the present study, which may be interpreted as a complex feedback mechanism working against DON-induced ferroptosis.

Furthermore, the in vitro study with IPEC-J2 cells confirmed that ferroptosis is involved in the DON-induced cell death in IPEC-J2 cells [8]. Specifically, DON decreased the viability of IPEC-J2 cells, while this change was alleviated by the supplementation of an iron chelator DFP, which is a potent inhibitor of ferroptosis. These outcomes revealed that ferroptosis might involve in the DON-mediated cell death. Furthermore, DON upregulated 4 ferroptosis-related genes (*DMT1*, *STEAP3*, *ACSL4* and *FTL*) [38–42]. Notably, the DFP treatment prevented or alleviated the changes on IPEC-J2 cell ACSL4 and FTL expression that was induced by DON. These outcomes further demonstrated that ferroptosis may be involved in the DON-induced cell death in the current study.

Because FTL was upregulated by DON in both the duodenum of piglets and IPEC-J2 cells, this study verified that DON mediates ferroptosis. Consistent with previous findings, DON reduced the viability of IPEC-J2 cells, while compensatory feedback for the upregulation of anti-ferroptosis protein FTL [43]. Notably, the present study showed that specific siRNA knockdown FTL protein production compromised the DON-induced cytotoxicity in IPEC-J2 cells. It is possible that FTL knockdown leads to an iron-rich response, which would lead to decelerated iron uptake and accelerated iron efflux, resulting in the decrease in the intracellular bioactive iron, and thus mitigating ferroptotic cell death in response to DON [13, 37]. However, the exact functions and mechanism of FTL in DON-induced ferroptosis need further exploration.

## Conclusions

In summary, the present study found that consumption of feed contaminated with ≥ 1.0 mg/kg DON caused piglet intestinal damage, as evidenced by changes in the histopathologic lesions and elevated serum LPS concentrations, presumably due to leaky gut. Meanwhile, the DON-induced intestinal injury was further evidenced by the impairment of redox homeostasis and ferroptosis signaling. Furthermore, DFP, a potent ferroptosis inhibitor, alleviated DON-induced cell death in IPEC-J2 cells in the present study. This result provided further evidence that ferroptosis might be involved in the DON-induced cell death. Moreover, specific siRNA knockdown FTL protein production compromised the DON-induced cytotoxicity in IPEC-J2 cells. Overall, these findings helped us better understand the toxicity of DON and provided novel target for the development remediation strategies to detoxify DON in piglets.

## Supplementary Information

**Additional file 1: Table S1**. Ingredients and nutrients composition of the Control diet.

**Additional file 2: Fig. S1**. Effects of DON (A) and DFP (B) on cell viability.

**Additional file 3: Table S2.** The sequences of siRNA for the knockdown analysis.

**Additional file 4: Table S3**. List of primers used for q-PCR analysis.

**Additional file 5: Table S4.** List of antibodies used for western blot analysis.

## Data Availability

The datasets used and/or analyzed during the current study are publicly available.

## Abbreviations

ACSL4: Acyl-CoA synthetase long chain family member 4
ALOX5: Arachidonate 5-lipoxygenase
ALOX12: Arachidonate 12-lipoxygenase, 12S type
ALOX15: Arachidonate 15-lipoxygenase
CISD1: CDGSH iron sulfur domain 1
DAO: Diamine oxidase
DMT1: Divalent metal transporter 1
DON: Deoxynivalenol
FPN: Ferroportin
FSP1: Ferroptosis suppressor protein 1
FTL: Ferritin light chain
FTH1: Ferritin heavy chain 1
GPX4: Glutathione peroxidase 4
GSH: Glutathione
HSPB1: Heat shock protein family B (small) member 1
LPS: Lipopolysaccharide
MDA: Malondialdehyde
PC: Protein carbonyl
ROS: Reactive oxygen species
SLC7A11: Solute carrier family 7 member 11
SOD: Superoxide dismutase
STEAP3: Six-transmembrane epithelial antigen of prostate 3
T-AOC: Total antioxidant capacity
TFR1: Transferrin receptor
