# Supplementary material for: Ferroptosis is involved in deoxynivalenol-induced intestinal damage in pigs
Source: J Anim Sci Biotechnol. 2023 Mar 16;14:29. doi: 10.1186/s40104-023-00841-4 (PMC10018831; doi:10.1186/s40104-023-00841-4)
Supplement: Supplementary file 1 — Additional file 1: Table S1. Ingredients and nutrients composition of the Control diet. [file 40104_2023_841_MOESM1_ESM.docx]

**Table S1** Ingredients and nutrients composition of the Control diet^a^

| **Ingredients** | **Percentage, %** | **Nutrition component** | **Content** |
| --- | --- | --- | --- |
| Corn | 57.0 | Crude protein, % | 18.3 |
| Soybean meal | 15.0 | Digestible energy, MJ/Kg | 3433 |
| Extruded soybean | 5.0 | Lysine, % | 1.40 |
| Flour | 5.0 | Methionine, % | 0.45 |
| Whey powder | 5.0 | Threonine, % | 0.91 |
| Fish meal | 5.0 | Tryptophan, % | 0.26 |
| Spray-dried plasma protein | 1.0 | Calcium, % | 0.81 |
| Soybean oil | 2.0 | Total phosphorus, % | 0.67 |
| Acidifier | 0.4 | Available phosphorus, % | 0.49 |
| Salt | 0.4 | DON | - |
| Choline chloride | 0.2 | Aflatoxin B_1_ | - |
| Premix^b^ | 4.0 | Zearalenone | - |

^a^ “-” means lower than the detection limit of 250, 2 and 30 µg/kg for DON, aflatoxin B_1_ and zearalenone, respectively; Control, base diet; 1.0 DON, basal diet supplemented with 1.0 mg/kg DON; 3.0 DON, basal diet supplemented with 3.0 mg/kg DON. The measured DON concentration in the Control, 1.0 DON, 3.0 DON and 3.0 DON+A were -, 1.02 and 3.13 mg/kg, respectively

^b^ Premix provided per kg diet: Fe, 90 mg; Cu, 100 mg; Zn, 50 mg; Mn, 60 mg; I, 1.0 mg; Co, 0.8 mg; Se, 0.4 mg; VitA, 13,333 IU; VitD3, 333 IU; VitE, 100 mg; VitK_3_, 4 mg; VitB_1_, 6 mg; VitB_2_, 15 mg; VitB_6_, 7 mg; VitB_12_, 75 μg; Nicotinamide, 75 mg; pantothenic acid, 30 mg; *d*-biotin, 0.25 mg; and folacin, 2.5 mg
