# Supplementary material for: Ferroptosis is involved in deoxynivalenol-induced intestinal damage in pigs
Source: J Anim Sci Biotechnol. 2023 Mar 16;14:29. doi: 10.1186/s40104-023-00841-4 (PMC10018831; doi:10.1186/s40104-023-00841-4)
Supplement: Supplementary file 2 — Additional file 2: Fig. S1. Effects of DON (A) and DFP (B) on cell viability. [file 40104_2023_841_MOESM2_ESM.docx]

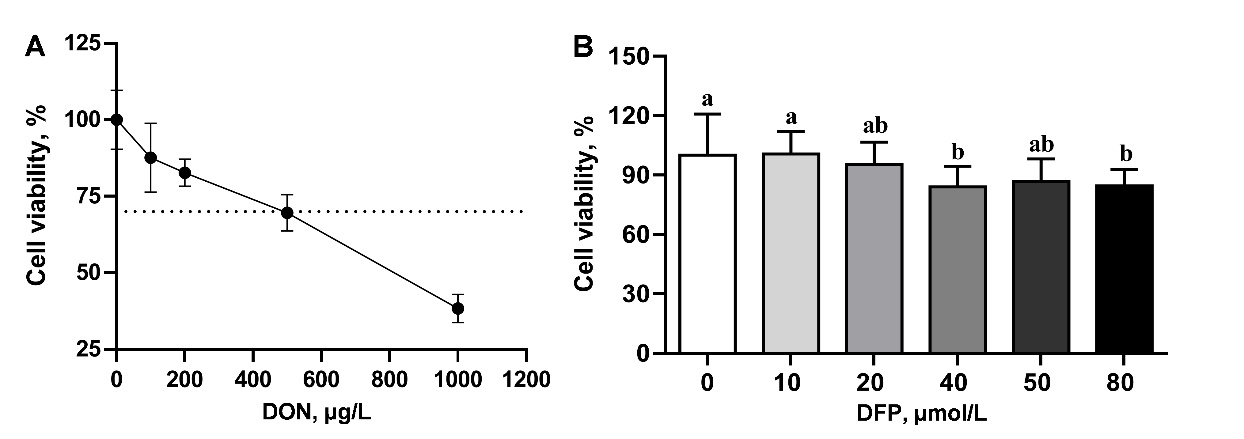


**Fig. S1** The cell viability of IPEC-J2 after 24h treatment with DON (A) and DFP (B). DON, μg/L; DFP, μmol/L. Values are expressed as means ± SD, *n* = 6. Labeled means without a common letter differ, *P <* 0.05
