# Supplementary material for: Ferroptosis is involved in deoxynivalenol-induced intestinal damage in pigs
Source: J Anim Sci Biotechnol. 2023 Mar 16;14:29. doi: 10.1186/s40104-023-00841-4 (PMC10018831; doi:10.1186/s40104-023-00841-4)
Supplement: Supplementary file 3 — Additional file 3: Table S2. The sequences of siRNA for the knockdown analysis. [file 40104_2023_841_MOESM3_ESM.docx]

**Table S2** The sequences of siRNA for the knockdown analysis

| **Oligonucleotides** | **Sense (5’to 3’ direction)** |
| --- | --- |
| Sus scrofa *FTL* siRNA159-181F | ACUCAUUGGUUCCUUUAAGGG |
| Sus scrofa *FTL* siRNA159-181R | CUUAAAGGAACCAAUGAGUCC |
| Negative control siRNA-F | UUCUCCGAACGUGUCACGUTT |
| Negative control siRNA-F | ACGUGACACGUUCGGAGAATT |
