# Supplementary material for: Ferroptosis is involved in deoxynivalenol-induced intestinal damage in pigs
Source: J Anim Sci Biotechnol. 2023 Mar 16;14:29. doi: 10.1186/s40104-023-00841-4 (PMC10018831; doi:10.1186/s40104-023-00841-4)
Supplement: Supplementary file 4 — Additional file 4: Table S3. List of primers used for q-PCR analysis. [file 40104_2023_841_MOESM4_ESM.docx]

**Table S3** List of primers used for q-PCR analysis^a^

| Gene name | Forward primer(5´to3´direction) | Reverse primer(5´to3´direction) |
| --- | --- | --- |
| *ACSL4* | GAGGGAGGCCATCGAGAATG | GACCAGGTGCTGGGATTTGT |
| *ALOX5* | TGAACTTCGGCCAGTACGAC | AGCTCGTTGTCCTGGAACTG |
| *ALOX12* | GGAGATCACTGCTCGGTACG | CTGGAGGGACACAGGGAAAC |
| *ALOX15* | TCCACTGGGTCGTCGTTCTA | ACTGAATTCCGTCTCCTTGCC |
| *CISD1* | ACACGTGCTTACCGATCGTC | CTGCGATCCACTCAACTCGT |
| *DMT1* | TCGTGTTTTACTTGGGTTGGCA | GGTGGCTTCTTCAGTCAGCA |
| *FTL* | CAGGCCTCCTACACCTACCT | TTCAAGAGACGCTCCGAACC |
| *FTH1* | AATTTGCGCTGCACGTGGT | TGGCTTTCACCTGCTCATGC |
| *FPN* | ACCATGTACCATGGATGGGTT | TCGTATTGTAGCATTCATATCTGC |
| *FSP1* | GAGGGAGGCCATCGAGAATG | GACCAGGTGCTGGGATTTGT |
| *GPX4* | TGGATGAAAGTCCAGCCCAAG | CTAGAGGTAGCACGGCAGGT |
| *HSPB1* | CACTCGAAAATACACGCTGCC | GGATGGTGATCTCTGCCGAC |
| *SLC7A11* | TCCGATCTTTGTTGCCCTCT | GGTCCCCAGAGAAGAGCATT |
| *STEAP3* | ACACTCACCTATGGCTGGAC | TCACACATGGCTCGTCTTCT |
| *TFR1* | TTGCCCAGATACTCTCCGAC | TGGCGGAAACCTTGAAGTTG |
| *β-actin* | CTCAAGTACCCCATCGAGCA | TCATCTTCTCACGGTTGGCT |

^a^ *ACSL4*, acyl-CoA synthetase long chain family member 4; *ALOX5*, arachidonate 5-lipoxygenase; *ALOX12*, arachidonate 12-lipoxygenase, 12S type; *ALOX15*, arachidonate 15-lipoxygenase; *CISD1*, CDGSH iron sulfur domain 1; *DMT1*, divalent metal transporter 1; *FTL*, ferritin light chain; *FTH1*, ferritin heavy chain 1; *FPN*, ferroportin; *FSP1*, ferroptosis suppressor protein 1; *GPX4*, glutathione peroxidase 4; *HSPB1*, heat shock protein family B (small) member 1; *SLC7A11*, solute carrier family 7 member 11; *STEAP3*, six-transmembrane epithelial antigen of prostate 3; *TFR1*, transferrin receptor
