# Supplementary material for: Ferroptosis is involved in deoxynivalenol-induced intestinal damage in pigs
Source: J Anim Sci Biotechnol. 2023 Mar 16;14:29. doi: 10.1186/s40104-023-00841-4 (PMC10018831; doi:10.1186/s40104-023-00841-4)
Supplement: Supplementary file 5 — Additional file 5: Table S4. List of antibodies used for western blot analysis. [file 40104_2023_841_MOESM5_ESM.docx]

**Table S4** List of antibodies used for western blot analysis^a^

| Antibody | Isotype | Dilution | Source |
| --- | --- | --- | --- |
| Primary antibody | | | |
| ACSL4 | Rabbit | 1:1000 | A6826 (ABclonal) |
| DMT1 | Rabbit | 1:1000 | A10231 (ABclonal) |
| FTH1 | Rabbit | 1:1000 | A1144 (ABclonal) |
| FTL | Rabbit | 1:1000 | A1768 (ABclonal) |
| FPN | Rabbit | 1:1000 | A14885 (ABclonal) |
| FSP1 | Rabbit | 1:1000 | 200886-AP (Proteintech) |
| STEAP3 | Rabbit | 1:1000 | A0683 (ABclonal) |
| β-Actin | Rabbit | 1:10,000 | AC026 (ABclonal) |
| Secondary antibody | | | |
| HRP Antibody | Goat anti rabbit | 1:10,000 | AS014 (ABclonal) |

^a^DMT1, metal transporter 1; ACSL4, acyl-coenzyme A synthetase long-chain family member 4; FTL, ferritin light chain; FTH1, ferritin heavy chain 1; STEAP3, six-transmembrane epithelial antigen of prostate 3; FSP1, ferroptosis suppressor protein 1
